# Effects of the fungicide metiram in outdoor freshwater microcosms: responses of invertebrates, primary producers and microbes

**DOI:** 10.1007/s10646-012-0909-0

**Published:** 2012-05-04

**Authors:** Ronghua Lin, Laura Buijse, Mauricio R. Dimitrov, Peter Dohmen, Sujitra Kosol, Lorraine Maltby, Ivo Roessink, Jos A. Sinkeldam, Hauke Smidt, René P. A. Van Wijngaarden, Theo C. M. Brock

**Affiliations:** 1 Alterra, Wageningen University and Research Centre, PO Box 47, 6700 AA, Wageningen, The Netherlands; 2 Institute for the Control of Agrochemicals, Ministry of Agriculture (ICAMA), Beijing, 100125 China; 3 Department of Animal and Plant Sciences, The University of Sheffield, Sheffield, S10 2TN UK; 4 Laboratory of Microbiology, Wageningen University, Dreijenplein 10, 6703 HB Wageningen, The Netherlands; 5 BASF SE, Agricultural Center, APD/EE-L1425, Speyerer Str. 2, 67117 Limburgerhof, Germany; 6 Thailand Institute of Scientific and Technological Research, 35 Moo 3, Tambon Klong Five, Klong Laung, Pathum Thani 12120 Thailand

**Keywords:** Model ecosystem, Aquatic risk assessment, Pesticide, Community effects, Population responses

## Abstract

The ecological impact of the dithiocarbamate fungicide metiram was studied in outdoor freshwater microcosms, consisting of 14 enclosures placed in an experimental ditch. The microcosms were treated three times (interval 7 days) with the formulated product BAS 222 28F (Polyram^®^). Intended metiram concentrations in the overlying water were 0, 4, 12, 36, 108 and 324 μg a.i./L. Responses of zooplankton, macroinvertebrates, phytoplankton, macrophytes, microbes and community metabolism endpoints were investigated. Dissipation half-life (DT_50_) of metiram was approximately 1–6 h in the water column of the microcosm test system and the metabolites formed were not persistent. Multivariate analysis indicated treatment-related effects on the zooplankton (NOEC_community_ = 36 μg a.i./L). Consistent treatment-related effects on the phytoplankton and macroinvertebrate communities and on the sediment microbial community could not be demonstrated or were minor. There was no evidence that metiram affected the biomass, abundance or functioning of aquatic hyphomycetes on decomposing alder leaves. The most sensitive populations in the microcosms comprised representatives of Rotifera with a NOEC of 12 μg a.i./L on isolated sampling days and a NOEC of 36 μg a.i./L on consecutive samplings. At the highest treatment-level populations of Copepoda (zooplankton) and the blue-green alga *Anabaena* (phytoplankton) also showed a short-term decline on consecutive sampling days (NOEC = 108 μg a.i./L). Indirect effects in the form of short-term increases in the abundance of a few macroinvertebrate and several phytoplankton taxa were also observed. The overall community and population level no-observed-effect concentration (NOEC_microcosm_) was 12–36 μg a.i./L. At higher treatment levels, including the test systems that received the highest dose, ecological recovery of affected measurement endpoints was fast (effect period < 8 weeks).

**Electronic supplementary material:**

The online version of this article (doi:10.1007/s10646-012-0909-0) contains supplementary material, which is available to authorized users.

## Introduction

This paper deals with the ecological impact of environmentally realistic concentrations of the dithiocarbamate fungicide metiram on freshwater organisms in outdoor freshwater microcosms. Despite the frequent use of fungicides to protect crops from fungal infections and the reported pollution of surface waters with these chemicals (e.g. Verro et al. 2009; Schäfer et al. 2011), relatively little experimental information is available on the ecological impact of realistic fungicide exposures on freshwater communities. The aquatic semi-field studies with fungicides published in the open literature are limited to chlorothalonil (Ernst et al. 1991), pentachlorophenol (e.g. Willis et al. 2004), carbendazim (Cuppen et al. 2000; Van den Brink et al. 2000; Slijkerman et al. 2004; Daam et al. 2009), triphenyltin (Roessink et al. 2006), fluazinam (Van Wijngaarden et al. 2010) and azoxystrobin (Gustafsson et al. 2010), illustrating the lack of open domain ecosystem-level information on effects of dithiocarbamates in edge-of-field surface waters.

The semi-field studies mentioned above focussed on measurement endpoints related to responses of primary producers and invertebrates, while treatment-related responses on aquatic fungi and bacteria hardly received attention. However, other ecotoxicological studies (also not including metiram) have demonstrated effects of fungicide exposure on aquatic fungi (e.g. Bundschuh et al. 2011; Dijksterhuis et al. 2011) and aquatic bacteria (e.g. Widenfalk et al. 2008; Milenkovski et al. 2010). Microbial communities are pivotal for the functioning of practically any ecosystem on Earth and that is why studying the potential effects of environmentally realistic pesticide exposures on the ecosystem services provided by microbes is important (Nienstedt et al. 2012). On the other hand, it is reported that functional redundancy and recovery potential of microbial communities may be high (Van den Brink et al. 2007). Here we begin to address this knowledge gap by investigating the effects of metiram on leaf litter breakdown and associated fungi (fungal biomass and hyphomycete abundance) and on the composition of the microbial community in the sediment compartment.

Formulated products of metiram have been used worldwide for over 40 years on a variety of fruits, vegetables, and ornamental crops to control fungal diseases like early blight, brown spot and black spot (McMullen and Jong 1971; Vawdrey et al. 2008; Horsfield et al. 2010). Metiram may unintentionally enter edge-of-field surface waters by, for example, spray drift. Metiram can be applied repeatedly (three up to nine times; minimum interval of 7 days) in crop protection programmes, potentially resulting in repeated short-term exposures in edge-of-field surface water of approximately 0.28–25 μg a.i./L (European Commission 2005; based on FOCUS calculations, personal communication with Peter Dohmen of BASF).

Acute laboratory toxicity data for standard and additional aquatic species and exposed to metiram demonstrate that the L(E)C50 values for fish, aquatic invertebrates and algae are 333–>20,000, 110–>1,000 and 63–>1,000 μg a.i./L, respectively (European Commission 2005). On average algae are more sensitive than aquatic invertebrates, but the difference in geometric mean L(E)C50 value for these taxonomic groups is less than a factor of 10. For metiram, Maltby et al. (2009) calculated a median HC5 (=hazardous concentration to 5 % of the tested species) of 40 μg a.i./L on basis of a species sensitivity distribution curve constructed with acute toxicity data for aquatic algae and aquatic invertebrates.

The aim of this paper is to evaluate the population and community level effects of realistic exposures of metiram in experimental freshwater ecosystems simulating the community of drainage ditches. This paper has its focus on treatment-related responses of zooplankton, macroinvertebrates, phytoplankton and microbes, as well as decomposition and community metabolism endpoints [e.g. dissolved oxygen (DO), pH].

## Materials and methods

### Test systems

The test was performed by using 14 enclosures (outdoor microcosms) situated in one of the experimental ditches located at the Sinderhoeve Experimental Station, Renkum, the Netherlands (Drent and Kersting 1993). Each enclosure consisted of a polycarbonate, translucent cylinder (diameter: 1.05 m; surface area 0.865 m^2^; height: 0.9 m), pushed approximately 0.15 m into the sandy loam sediment of the ditch. Water depth was approximately 0.5 m. The enclosures were installed into the experimental ditch on 16 July 2010, 25 days before start of the treatment. The enclosures simulated a shallow, mesotrophic, macrophyte-dominated freshwater community, but fish was not present. Shortly after placing the enclosures in the ditch, 30 individuals of *Gammarus pulex* and 28 individuals of *Asellus aquaticus* were introduced in each enclosure to ensure a more or less equal distribution in each test system. This was done since these macroinvertebrate shredders play an important role in the breakdown of leaf material. Both species are common in the experimental ditches of the Sinderhoeve Research Station, but their densities appeared to be low in the ditch selected for the experiment.

### Fungicide treatment

Metiram was applied to enclosures as the formulated product BAS 222 28F (Polyram^®^) that was provided by BASF SE and had a measured active ingredient content of 70.39 % (w/w). The test substance was applied on the 10th, 17th and 24th of August, 2010 (interval 7 days) and the intended initial metiram concentrations in the overlying water of the enclosures were 0, 4, 12, 36, 108, and 324 μg a.i./L, in line with the expected population level effects on basis of single species toxicity tests and predicted environmental concentrations (PECs). The lowest test concentration is based on the reported 21-day NOEC of *Daphnia magna* (4.3 μg a.i./L) and the highest test concentration on the (lower) acute toxicity values reported for Tier-1 aquatic standard test species (see introduction section). Four test systems were used as controls and two test systems were used for each metiram concentration. Treatments and controls were assigned randomly to the 14 enclosures. The fungicide was applied by pouring approximately 2 L of dosing solution over the water surface and gently stirring to mix the compound in the water column. The control enclosures received water only.

### Fungicide residues in water

Since metiram shows an extremely fast dissipation in water, actual peak concentrations were estimated by analyzing the metiram concentration of the dosing solutions and measuring the exact volume of dosing solution applied to each enclosure. In addition, 2 h after each application duplicate 500 mL depth-integrated samples were taken from each enclosure by means of a vacuum pump and stainless steel suction tubes and stored in borosilicate glass flasks. Metiram analysis of these water samples was performed at Alterra by liquid chromatography with tandem mass spectrometric detection by measuring the concentration of the ethylenebisdithiocarbamate (EBDC) ligand released in the water samples. The formed EBDC analyte was methylated with iodomethane. The methylated EBDC was then quantified by HPLC with MS/MS detection. The limit of quantification (LOQ) of the method for metiram analysis in water is 50 ng/L.

In addition, water samples collected on day 17 (3 days after the last metiram application) and on day 59 (end of experiment) from enclosures that received the three highest treatment levels were analysed by BASF SE for concentrations of metiram and its degradates ethylene–thiourea (ETU), ethylene–urea (EU), hydantoin (HY), carbamid, ethylene bisisothiocarbamate (EBIS) and C_8_H_8_N_4_S_2_ (TDIT). Before analysis of these compounds by means of LC/MS/MS, metiram water samples were methylated with iodomethane. Samples for the analysis of EBIS, carbamid and TDIT were acidified with formic acid, and samples for ETU, EU and HY measurements with acetic acid.

### Ecological endpoints investigated

The endpoints measured in this study are summarized in Table 1. Artificial substrates, in the form of litter bags and pebble baskets, were used to monitor the macroinvertebrate community. Two litter bags (initially containing 2 g dry weight of *Populus* leaves) and two pebble baskets were incubated in the enclosures for approximately 2 weeks prior to sampling. For a detailed description of the artificial substrates and the collection of macroinvertebrates see Brock et al. (1992).

**Table 1:** Summary of endpoints investigated in microcosm study

Endpoint	Unit	Sampling days
*Macroinvertebrates*		
Species composition	Taxa richness	−12, 15, 29, 43, 57
Abundance	Numbers/L	
*Zooplankton*		
Species composition	Taxa richness	−1, 3, 10, 17, 24, 31, 48,59
Abundance	Numbers/L	
*Phytoplankton*		
Species composition	Taxa richness	−1, 3, 10, 17, 24, 31, 48, 59
Abundance	Numbers/mL	
Chlorophyll *a*	μg/L	
*Macrophytes*		
Above ground biomass	g dry weight/enclosure	−14, 62
*Microbes and decomposition*		
Fungal biomass on leaves	μg fungi/mg freeze-dried leaf	−4, 3, 10, 17, 31, 52
Fungal species abundance on leaves	Conidia production score	−4, 3, 10, 17, 31, 52
Leaf decomposition	g dry weight (mass loss)	−4, 3, 10, 17, 31, 52
Sediment bacterial and fungal community structure	Presence and intensity of bands in the DGGE profile	−4, 3, 10, 17, 24, 31, 48, 59
*Physico*-*chemical*		
pH, DO, temp., EC	–, mg/L, °C, μs/cm	−1, 3, 10, 17, 24, 31, 48, 59
Alkalinity	meq/L	−1, 17, 59
Nutrients	mg/L	−1, 59

*DO* dissolved oxygen, *EC* electrical conductivity

Approximately 10 L depth-integrated water samples were collected from each enclosure by means of a perspex tube to monitor the plankton community. A 5-L aliquot of each sample was filtered through a 55-μm mesh net and the collected zooplankton removed and preserved with acetate buffered lugol solution. The remaining 5-L aliquot of each sample was filtered through a 20-μm mesh net and the collected phytoplankton removed and also preserved with acetate buffered lugol solution. The filtered water was returned to its original enclosure. Cladocerans, ostracods and copepods were counted using a binocular microscope at a magnification of 25 times. Using an inverted microscope (100–400 times magnification), the numbers of rotifers and copepod nauplii were determined by counting the specimens in a known volume. Rotifers and cladocerans were identified to the lowest practical taxonomic level (in most cases species/genus level). Copepods (except nauplii) were divided into calanoids and cyclopoids.

Phytoplankton species composition was studied by counting the number of cells or colonies in a known volume of concentrated sample. Taxa and abundance measures were based on a maximum of 40 counting fields or a minimum of 200 observations (in at least 20 counting fields of a cuvette) using an inverted microscope (magnification ×400). Phytoplankton were identified to the lowest practical taxonomic level. Concentrations of total phytoplankton chlorophyll *a* were measured by means of a BBE AlgaeTorch (Envitech Ltd.). The measurements with this instrument are based on the natural fluorescence of algae cells as described for the BBE spectrofluorometric probe by Beutler et al. (2002). The BBE AlgaeTorch was placed in the bucket that contained 10 L of depth-integrated water sample from each enclosure and chlorophyll *a* measurements were performed at least three times in each sample.

Pre-application above-sediment macrophyte biomass was assessed by sampling three representative plots (0.25 m^2^) inside the study ditch but outside of the enclosures. The above-sediment macrophyte biomass was sampled from each enclosure at the end of the study. Sampled plant material was rinsed under tap water to remove loosely attached materials (e.g. sediment particles and invertebrates), packed in pre-weighted aluminium foil and dried in an oven for at least 48 h at 70 °C and until constant weight was reached.

Temperature, pH and DO were measured at approximately 25 cm water depth using an HQ40D (Hach) oxygen-acidity meter, equipped with a luminescent DO probe. Electrical conductivity was measured at the same water depth using a WTW LF191 meter and the alkalinity of a 100-mL unfiltered water sample from each enclosure was measured by titration with 0.02 N HCl to pH 4.2. Nutrient status was assessed by filtering a 100-mL depth integrated water sample through a GF/C glass fibre filter (mesh size 1.2 μm). The filtrate was stored in polyethylene flasks at −18 °C until analysis of total nitrogen, nitrate/nitrite, ammonium, ortho-phosphate and total phosphate following standard procedures.

Fungal biomass, aquatic hyphomycete abundance and leaf decomposition were assessed by deploying 5 or 8 g of air-dried alder leaf material in fine mesh (600 μm) or coarse mesh (0.5 cm × 0.5 cm) bags. The fine mesh excluded macroinvertebrates whereas the coarse mesh did not. Leaf material was conditioned in an experimental ditch for 4 weeks and allocated to the enclosures 4 days before the first fungicide application. Fifteen fine and 15 coarse mesh bags were initially placed in each enclosure and three bags of each type were retrieved on each sampling date. After retrieval, all leaf material was washed gently to remove attached sediment before processing.

Twenty-five 1-cm diameter alder leaf discs were cut per fine mesh bag; 10 discs being used for fungal identification and 15 discs for fungal biomass measurements. Conidial morphology was used to identify aquatic hyphomycetes (Ingold 1975) and leaf discs were agitated in sterile distilled water for 4 days prior to examination to stimulate sporulation. A measure of relative abundance was obtained by assigning species a score between 0 and 4 based on conidial abundance: 0 (conidia absent), 1 (1–3 conidia), 2 (4–9 conidia), 3 (10–15 conidia), 4 (>15 conidia). The remaining 15 leaf discs were placed in sterile eppendorf tubes and stored frozen until analysed for fungal biomass using an ergosterol assay modified from Newell and Fell (1992).

Alder leaf decomposition, expressed as mass loss, was determined for leaf material deployed in fine mesh bags (i.e. microbial decomposition) and coarse mesh bags (i.e. microbial decomposition + invertebrate consumption). Mass loss calculations took account of leaf material used for the assessment of fungal biomass and species identification.

In order to study the effects of metiram application on microbes in the sediment, three cores of the upper 3 cm of sediment (using a Perspex tune with an inner diameter of 2.4 cm) from each control enclosure and from those that received the two highest treatment levels (108 and 324 μg metiram/L) were collected on each sampling date (see Table 1). The three cores were thoroughly mixed in order to get one homogeneous sediment sample per test system. Subsequently, subsamples were taken and stored in eppendorf tubes, which were directly put on dry ice and later stored at −80 °C until use. Microbial community structure was assessed using molecular techniques (Bending et al. 2007; Ferreira et al. 2009; Tzeneva et al. 2008; Villeneuve et al. 2011). Total DNA was isolated from these subsamples using the FastDNA^®^ Kit for Soil (MP Biomedicals, Santa Ana, CA) according to the manufacture’s protocol (Mincer et al. 2005). Polymerase chain reaction (PCR) (Mullis et al. 1986) amplifications were performed using the isolated DNA where the bacterial community was targeted by amplification of 16S rRNA gene fragments, whereas fungal community was targeted by both amplification of 18S rRNA gene fragments and internal transcribed spacer (ITS) region fragments, using the primers listed in Table 2. PCR products were analysed by denaturating gradient gel electrophoresis (DGGE) using a Dcode Universal Mutation Detection System (Bio-Rad, Hercules, CA, USA) (Muyzer et al. 1993). DGGE was performed on polyacrylamide gels with a denaturant gradient from 30 to 60 % for the separation of 16S rRNA gene amplicons, from 20 to 45 % for 18S rRNA gene amplicons and from 20 to 50 % for the ITS region (100 % denaturing acrylamide was defined as 7 M urea and 40 % (v/v) formamide). Aliquots of PCR products were loaded on the gel and electrophoresis was carried out with 1 × Tris-acetate-EDTA buffer at 60 °C and at 85 V for 16 h. After the completion of the electrophoresis, gels were silver-stained (Sanguinetti et al. 1994) and scanned. DGGE band detection and quantification of band intensity were performed using the Bionumerics software version 4.61 (Applied Maths, Belgium) (Tzeneva et al. 2009) and the results used to assess operational taxonomic units (OTUs) for microbes in the sediment samples (Massana and Jürgens 2003).

**Table 2:** Primers used to assess OTUs for bacteria and fungi present in sediment samples

Primer	Sequence 5′–3′	Specificity	References
F968-GC	CGCCCGGGGCGCGCCCCGGGCGGGGCGGG GGCACGGGGGGAACGCGAAGAACCTTAC	Bacteria	Nubel et al. (1996)
1401R	CGGTGTGTACAAGACCC	Bacteria	Nubel et al. (1996)
NS1	GTAGTCATATGCTTGTCTC	Fungi	White et al. (1990)
GCfung	CGCCCGCCGCGCCCCGCGCCCGGCCCGCCGCCCCCGCCCCATTCCCCGTTACCCGTTG	Fungi	May et al. (2001)
ITS3-GC	CGCCCGCCGCGCCCCGCGCCCGGCCCGCCGCCCCCGCCCCGCATCGATGAAGAACGCAGC	Fungi	White et al. (1990)
ITS4	TCCTCCGCTTATTGATATGC	Fungi	White et al. (1990)

### Statistics

Prior to statistical analysis, zooplankton, macroinvertebrate, phytoplankton, bacteria and fungi data were Ln(*ax* + 1) transformed, where *x* stands for the abundance value. For zooplankton *a* = 10, for macroinvertebrates *a* = 2, for phytoplankton *a* = 1.47, for bacteria *a* = 12.5 and for fungi *a* = 20. This was done to down-weigh high abundance values and to approximate a normal distribution for the data (for rationale see Van den Brink et al. 2000). NOEC calculations at taxon or parameter level (*p* ≤ 0.05) were carried out using the Williams test (ANOVA; Williams 1972). The analyses were performed with the Community Analysis computer program (Hommen et al. 1994).

Effects on the zooplankton, macroinvertebrate, phytoplankton, sediment bacterial and sediment fungal communities were analysed by the principle response curves (PRC) method (Van den Brink and Ter Braak 1999). In addition to the overall significance of the effects of the treatment regime (Monte Carlo permutation tests), each treatment was also compared to the controls to identify the NOEC at the community level. The NOEC calculations were carried out by applying the Williams test to the sample scores of the first principle component of each sampling date in turn (Van den Brink et al. 1996). Effects were considered consistent when they showed statistically significant deviations pointing in the same direction for at least two consecutive sampling points or occurred on a single sampling day during or immediately after the application period. The data were also evaluated for possible artefacts relating to small magnitude of measured counts, or having no treatment related concentration–response and/or no clear causality with community interactions or timing (European Commission 2002).

## Results

### Physico-chemical measurements

Water temperatures in the enclosures were approximately 18–19 °C at the start of the experiment and during the metiram application period. Temperatures gradually declined from sampling day 17 onwards and the lowest water temperature measured in the enclosures was approximately 12 °C. Data on weather conditions during the experiment can be found in the Supporting Information.

Statistically significant changes in physico-chemical endpoints are presented in Table 3 and temporal trends are presented in the Supporting Information. A significant, but small, treatment-related increase in electronic conductivity was observed at the highest treatment-level on the days 10 and 17. Control enclosure pH values ranged between 7.2 and 9.0 and pH of control enclosures were significantly lower than that of treatment enclosures in the pre-treatment period (day −1) and immediately after first metiram application (day 3). However, on sampling days 10, 17 and 48, pH values showed a small, but statistically significant, treatment-related decrease and on day 59 there was a significant increase in pH although all deviations were less than 1 pH unit.

**Table 3:** NOECs (Williams test, *p* < 0.05) in μg a.i./L (expressed in terms of nominal treatment level) for physico-chemical characteristics observed on each sampling date in the metiram enclosure experiment

Endpoint	Day after first application								Note
	−1	3	10	17	24	31	48	59	
EC	–	108↑^a^	108↑^a^	–	–	–	–	36↓^a^	SI Fig. I-A
pH	4↑^a^	4↑^a,b^	108↓^a^	<4↓^a^	–	–	12↓^a^	<4↑^a^	SI Fig. I-B
O_2_	–	–	–	108↓^a^	–	–	<4↑^a^	–	SI Fig. I-C
Alkalinity	–			<4↑^a^				12↓^a^	SI Fig. I-D

↓ = decrease, ↑ = increase, – = no significant effect (Williams test, *p* > 0.05). *SI* Supporting Information

^a^Quantitatively small difference relative to controls

^b^Downward trend relative to pre-treatment (day −1)

DO concentrations were relatively low in all enclosures during the application period, but were always higher than 4 mg/L. After day 17, DO levels increased to approximately 10 mg/L. A small but significant decline in DO was observed on day 17 in the 324 μg a.i./L treatment, and on day 48 DO levels were significantly higher in all treated enclosures relative to controls. All treatment-related differences in DO concentration were less than 1–2 mg/L.

Alkalinity values in control enclosures ranged between 1.08 to 1.24 mmol/L. On day 17, a small but significant treatment-related increase in alkalinity was measured while at the end of the experiment (day 59) a small but significant treatment-related decrease was observed.

At the start and the end of the experiment, nitrate/nitrite, ammonium, ortho-phosphate and total phosphate concentrations in depth-integrated water samples from the enclosures were below detection limits. On days −1 and day 59 measured concentrations of total soluble nitrogen ranged between 0.9–1.1 and 0.4–0.7 mg/L, respectively. Treatment-related effects on nutrient concentrations in the water column could not be demonstrated.

### Exposure concentrations

Metiram concentrations in the dosing solutions were on average 92.7 % of the intended concentration (range of 79.0–113.4 %), but concentrations in depth-integrated water samples collected approximately 2 h after the first fungicide application were only 36.6 % (range 16.0–65.1 %) of the initial concentration, highlighting the rapid disappearance of metiram from the water compartment. Unfortunately, the water samples collected 2 h after the second and third treatment were lost due to technical problems during metiram analysis (corrosion of the metal tubes of the measurement equipment).

Three days after the last application (day 17), the average concentration in water samples collected from the 324 μg metiram/L enclosures was 0.14 μg metiram/L (0.04 % of the initial concentration) and no metiram was detected in samples from the 108 and 36 μg metiram/L enclosures (<0.05 μg metiram/L). Average concentrations of the metabolites EU and ETU in day 17 water samples from the 324, 108 and 36 μg metiram/L enclosures, were 38.8 μg EU/L and 12.2 μg ETU/L, 15.6 μg EU/L and 0.6 μg ETU/L and 4.3 μg EU/L and 0.13 μg ETU/L, respectively. All other degradates analysed were below detection limits (i.e. <20 μg/L for HY; <0.2 μg/L for EBIS, carbimid and TDIT). At the end of the experiment (day 59 after the first treatment) the concentrations of metiram and all metabolites analysed were below detection limits (metiram < 0.05 μg/L; EU < 1.0 μg/L; HY < 20 μg/L; ETU, EBIS, carbimid and TDIT < 0.2 μg/L). These data illustrate that metiram dissipates very fast (estimated water dissipation DT50 of approximately 1–6 h) and that its metabolites are not persistent in the water compartment.

### Zooplankton responses

**Fig. 1:**
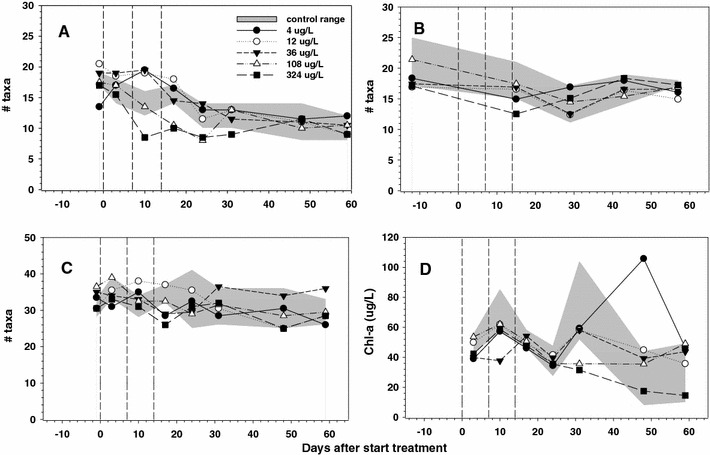
Dynamics in taxa richness of zooplankton (**a**), macroinvertebrates (**b**), phytoplankton (**c**) and chlorophyll *a* biomass of phytoplankton (**d**) in the different treatments of the metiram enclosure experiment. The *shaded area* shows the range observed in control enclosures and the geometric mean values are presented per treatment. The *vertical dotted lines* indicate days of metiram application. The NOECs for treatment-related responses are presented in Tables 4, 5 and 6

Of the 30 zooplankton taxa collected during this study, 23 were rotifers, three were cladocerans, three were copepods and one was an ostracod. The most abundant zooplankton taxa in decreasing order were: *Anuraeopsis fissa* (Rotifera), copepod nauplii (Copepoda), *Polyarthra remata* (Rotifera), *Trichocerca* gr*. similis* (Rotifera), *Keratella cochlearis* (Rotifera), *Lecane* gr*. luna* (Rotifera), Cyclopoida (Copepoda), *Ceriodaphnia* sp. (Cladocera), *Trichocerca* gr*. porcellus* (Rotifera) and *Squatinella rostrum* (Rotifera). The number of zooplankton taxa was significantly reduced relative to controls at the highest dose (324 μg a.i./L) 10 days after the first application (Table 4). However, a statistically non-significant decline in zooplankton richness was observed in the post-exposure period (days 17–24) in the two highest doses (Fig. 1a).

**Table 4:** NOECs (Williams test, *p* < 0.05) in μg metiram/L (expressed in terms of nominal treatment level) for zooplankton community and individual taxa that showed a treatment-related effect on at least one sampling

		Day after first application								Note
		−4	3	10	17	24	31	48	59	
Zooplankton	Total taxa richness	–	–	108↓	–	–	–	–	–	Figure 1a
	Community	–	108	36	36	36	–	–	–	Figure 2a
Taxon group	Taxon name									
Rotifera	Total abundance	–	36↓	36↓	36↓	–	–	–	–	Figure 3a
	*A. fissa*	–	36↓	36↓	36↓	36↓	–	–	–	Figure 3b
	*Cephalodella gibba*	108↑	–	–	–	–	–	–	–	Low density*
	*K. cochlearis*	–	108↓	108↓	36↓	12↓	–	–	–	
	*Lepadella patella*	–	–	–	–	–	–	–	12↑	Low density
	*P. remata*	–	12↓	36↓	36↓	12↓	36↓	–	–	Figure 3c
	*S. longicaudum*	–	–	108↓	36↓	–	108↓	–	–	
	*S. rostrum*	–	108↓	–	–	–	–	–	–	
	*T.* gr*. similis*	–	108↓	108↓	36↓	–	–	–	–	Figure 3d
	*Trichotria pocillum*	–	–	–	–	–	36↑	–	–	Low density
Copepoda	Total abundance	–	108↓	108↓	108↓	12↓	–	108↓	–	Figure 3e
	Calanoida	108↑	–	–	–	–	–	–	–	
	Cyclopoida	–	108↓	108↓	36↓	108↓	108↓	–	–	Figure 3f
	Nauplii	–	108↓	108↓	–	12↓	–	108↓	–	
Cladocera	Total abundance	–	–	–	–	–	–	–	–	Figure 3g
	*Alona* sp.	–	–	–	–	–	–	108↑	–	Low density
Ostracoda	Ostracoda	–	–	–	108↑	–	–	–	–	Low density

↓ = reduction in abundance, ↑ = increase in abundance, – = no significant effect (Williams test, *p* > 0.05)

* Low density means that the number of individuals per sample was on average <10 individuals/L when the statistically significant difference was observed

**Fig. 2:**
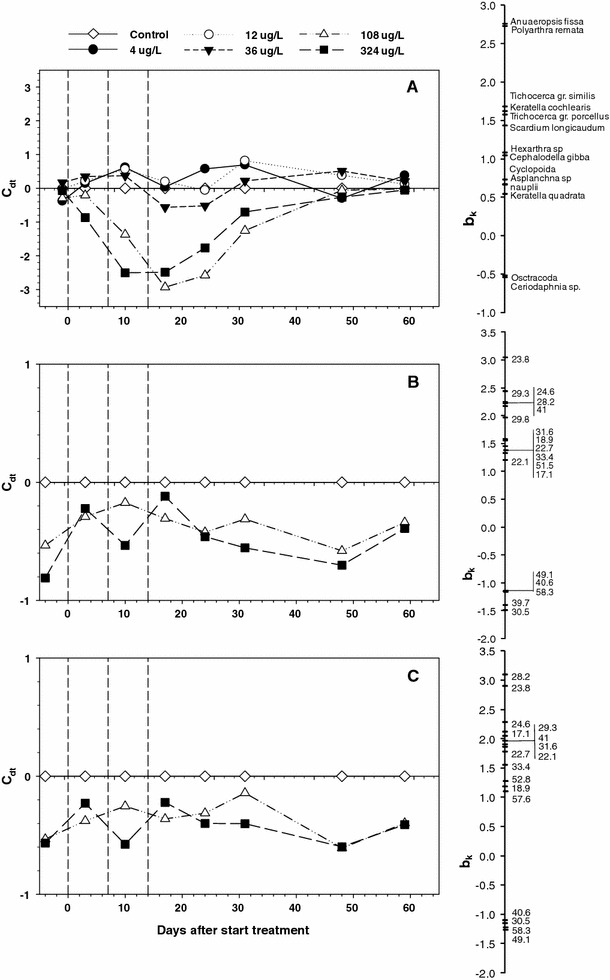
Principal response curve diagram for the zooplankton dataset (**a**), the sediment bacteria DGGE band intensity dataset (**b**) and sediment bacteria OTUs dataset based on presence of DGGE bands (**c**) of the metiram enclosure study (for further explanation see description in text). The *vertical dotted lines* indicate days of metiram application. *C*_*dt*_ canonical coefficient showing the difference between treatments and control in time, *b*_*k*_ species weight that indicates the affinity of the taxon (**a**) or specific DGGE bands on the gels (**b**, **c**) with the PRC. The NOECs for treatment-related responses are presented in Tables 4 and 7. **a** 33 % of all variance could be attributed to sampling date (*horizontal axis*) and 31 % to treatment level, 34 % of which is displayed on the *vertical axis*. **b** 39 % of all variance could be attributed to sampling date and 21 % to treatment level, 17 % of which is displayed on the *vertical axis*. **c** 35 % of all variance could be attributed to sampling date and 23 % to treatment level, 17 % of which is displayed on the *vertical axis*

Multivariate PRC analysis indicated that the zooplankton community was significantly affected by exposure to metiram (Monte Carlo permutation test *p* = 0.009) with the rotifers *Anureopsis fissa* and *P. remata* being particularly negatively affected by the metiram application (Fig. 2a). Significant treatment-related effects on the zooplankton community were detected at the highest concentration (324 μg a.i./L) on day 3 and in the 108 and 324 μg a.i./L enclosures on days 10, 17 and 24 (Table 4).

**Fig. 3:**
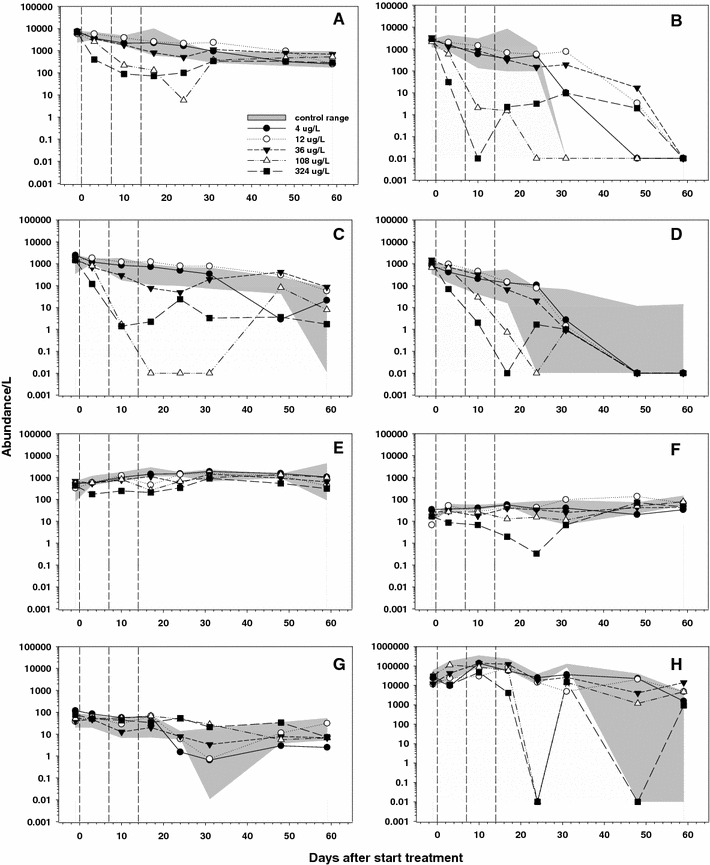
Dynamics in population abundance of zooplankton taxa (**a**–**g**) and of the phytoplankton taxon *Anabaena* sp. (**h**) in the different treatments of the metiram enclosure experiment. The *shaded area* shows the range observed in control enclosures and the geometric mean values are presented per treatment. **a** Total Rotifera, **b** *A. fissa* (Rotifera), **c** *P. remata* (Rotifera), **d** *T.* gr. *similis* (Rotifera), **e** total Copepoda, **f** Cyclopoida (Copepoda), **g** nauplii (Copepoda), **h** *Anabaena* sp. (Cyanophyta). The NOECs for treatment-related responses are presented in Tables 4 (zooplankton) and 6 (*Anabaena* sp.)

At the population level, statistically significant differences between treatments and controls could be observed for 13 of the 30 zooplankton taxa, but for two of them these differences occurred in the pre-treatment period (Table 4) and consequently were not treatment-related. Results of univariate analyses of population data (Williams test, *p* < 0.05) are presented in Table 4 and temporal trends illustrated in Fig. 3.

Treatment-related effects on Rotifera total abundance were observed at the two highest treatment-levels (NOEC = 36 μg a.i./L) and started soon after first application. Full recovery was observed on day 31 (Fig. 3a). Similar treatment-related declines were observed for *A. fissa* from day 3 to day 24 at the two highest treatment levels (NOEC_population_ = 36 μg a.i./L). After day 31 densities in controls declined to zero and differences in abundance between treatments did not show a clear concentration–response relationship (Fig. 3b; Table 4). *P. remata* was the most sensitive rotifer species with minor but significant declines observed at the 36 μg a.i./L treatment level (days 3 and 24) and clear treatment-related declines observed from day 3 to day 31 at 108 and 324 μg a.i./L, followed by recovery. Note, however, that the decline in the 108 μg a.i./L enclosures was more pronounced than in enclosures that received 324 μg a.i./L (Fig. 3c; Table 4). The abundance of the rotifers *T.* gr. *similis* and *K. cochlearis* declined in the highest treatment level from day 3 to day 17 and in the 108 μg a.i./L enclosures on day 17. After day 17, abundance of *T.* gr. *similis* declined in all enclosures, including the controls (Fig. 3d; Table 4). Significant declines in the abundance of *K. cochlearis* were observed in the 36 μg a.i./L enclosures on day 24 (Table 4). Treatment-related declines in abundance of the rotifer *Scaridium longicaudum* were observed on day 10 (NOEC = 108 μg a.i./L), day 17 (NOEC = 36 μg a.i./L) and day 31 (NOEC = 108 μg a.i./L) and at the highest treatment level this species was not detected from day 10 to day 31, followed by recovery (Table 4).

Effects of metiram on Copepoda total abundance were consistent, but small. Statistically significant treatment-related declines in abundance were observed from day 3 up to day 24 and on day 48 (NOECs of 108 μg a.i./L, except on day 24 when a NOEC of 12 μg a.i./L was calculated) (Fig. 3e; Table 4). Treatment-related declines in Cyclopoida abundance were observed from day 3 to day 31 at the highest treatment level (NOEC = 108 μg a.i./L), except on day 17 when a NOEC of 36 μg a.i./L could be calculated. Full recovery of Cyclopoida was observed after day 31 (Fig. 3f; Table 4). Copepod nauplii were abundant in all enclosures and minor, but statistically significant, declines were observed at the highest concentration (NOEC = 108 μg a.i./L) on days 3, 10, 24 and 48. On day 24, the calculated NOEC was 12 μg a.i./L (Table 4).

Total abundance of Cladocera was not affected by metiram (Fig. 3g; Table 4) and the only treatment-related response observed was for *Alona* sp. on day 48 when a NOEC of 108 μg a.i./L could be calculated (Table 4). All other populations of Cladocera did not show a treatment-related response. Ostracoda occurred at low densities in all enclosures and a significant increase in abundance was observed (Table 4; NOEC = 108 μg a.i./L) on an single sampling date (day 17).

### Macroinvertebrate responses

Sixty-three macroinvertebrate taxa were collected from the enclosures, the majority of which were Insecta (34 taxa), Mollusca (10), Oligochaeta (6), Hirudinea (5), Turbellaria (5), Crustacea (2) and Hydracarina (1). Several of these taxa occurred in low densities and/or were observed on a limited number of sampling dates only. The most abundant macroinvertebrate taxa in decreasing order were: *Dero* sp. (Oligochatea), *Chaoborus* sp. (Insecta), Chironomini (Insecta), *Mesostoma* sp. (Turbellaria), *Lumbriculus* sp. (Oligochaeta), Orthocladinae (Insecta), Ceratopogonidae (Insecta), *Caenis* sp. (Insecta), Zygoptera (Insecta) and *Dugesia lugubris* (Turbellaria).

A small decrease in the number of macroinvertebrate taxa relative to controls could be observed on day 15 (a day after the third metiram application) in the enclosures that received the highest concentration (324 μg a.i./L) (Fig. 1b; Table 5). Treatment-related effects of metiram on the macroinvertebrate community could not be demonstrated by means of multivariate PRC analysis (Monte Carlo permutation test *p* = 0.83). Although statistically significant differences between treatments and controls could be observed for 15 of the 63 macroinvertebrate taxa, these deviations predominantly occurred on isolated sampling days (Table 5). The only macroinvertebrate taxon for which statistical significant differences were observed on two consecutive samplings in the post-treatment period (days 43 and 57) was Dytiscidae larvae, but this taxon occurred in low densities (always <5 individuals per sample) and the effect concerned a treatment-related increase. For the ephemeropteran *Caenis* sp. and the mollusc *Gyraulus crista*, a statistically significant decline in numbers was calculated on day 15 (immediately after the third application), but densities of both taxa were low in all enclosures (Table 5).

**Table 5:** NOECs (Williams test, *p* < 0.05) in μg metiram/L (expressed in terms of nominal treatment level) for macroinvertebrate community and individual taxa that showed a treatment-related effect on at least one sampling

		Day after first application					Note
		−12	15	29	43	57	
Macro-invertebrates	Community	–	–	–	–	–	
	Total taxa richness	–	108↓	–	–	–	Figure 1b
Taxon group	Taxon name						
Crustacea	Asellidae	–	–	–	–	36↑	Low density^a^
Hirudinea	*Erpobdella* sp.	–	–	–	–	108↑	Low density
	*Helobdella stagnalis*	–	–	108↑	–	–	Low density
Insecta	Anisoptera	108↓	–	–	–	–	Low density
	*Caenis* sp.	–	36↓	–	–	–	Low density
	*Chironomini*	108↓	–	–	–	–	
	*Cloeon dipterum*	–	–	–	108↑	–	Low density
	Dytiscidae (larva)	–	–	–	108↑	108↑	Low density
	Haliplidae (larva)	–	–	–	108↑	–	Low density
	*Helophorus* sp.	–	–	–	108↑	–	Low density
	*Notonecta* sp.	–	–	–	–	108↑	Low density
	*Sigara* sp.	–	–	–	–	108↑	Low density
Mollusca	*G. crista*	–	108↓	–	–	–	Low density
	*Planorbis* sp.	36↓	–	–	–	–	Pre-treatment
Oligochaeta	Tubificidae	–	–	–	108↑	–	Low density

↓ = reduction in abundance, ↑ = increase in abundance; – = no significant effect (Williams test, *p* > 0.05)

^a^Low density means that the number of individuals per sample was <5

### Phytoplankton responses

One hundred and nine phytoplankton taxa were collected during this study, the majority of which were Chlorophyta (49 taxa), Desmidiaceae (23), Cyanophyta (14), Diatomeae (10), Euglenophyceae (7), Chrysophyceae (3), Dinoflagellata (2) and Cryptophyceae (1). A limited number of taxa dominated the phytoplankton community and many taxa occurred in low densities and/or were observed on a limited number of sampling dates only. The most abundant phytoplankton taxa in decreasing order were: *Volvox* (Chlorophyta), *Scenedesmus arcuatus* (Chlorophyta), *Tetraedron minimum* (Chlorophyta), Pennales (Diatomeae), Pseudanabaenaceae (Cyanophyta), *Phacotus lendneri* (Chlorophyta), *Aphanocapsa* (Cyanophyta), *Anabaena* (Cyanophyta), *Oocystis* (Chlorophyta) and *Aphanothece* (Cyanophyta).

A small decrease in the number of phytoplankton taxa relative to controls was observed on day 17 at the highest concentration (324 μg a.i./L) (Fig. 1c; Table 6). There was little evidence of a treatment-related response in total chlorophyll *a* biomass with significant reductions only observed on day 31 at the highest concentration (Fig. 1d; Table 6).

**Table 6:** NOECs (Williams test, *p* < 0.05) in μg metiram/L (expressed in terms of nominal treatment level) for phytoplankton community and individual taxa

		Day after first application								Note
		−1	3	10	17	24	31	48	59	
Phytoplankton	Community	–	–	–	–	–	–	–	–	
	Total taxa richness	–	–	–	108↓	–	–	–	–	Figure 1c
	Chlorophyll *a*	–	–	–	–	–	108↓	–	–	Figure 1d
Taxon group	Taxon name									
Chlorophyta	Total abundance	–	–	–	–	–	–	–	–	SI Fig. II-A
	Chlorophyta colony	–	–	–	–	–	36↑	–	–	
	Chlorophyta loose cells	–	–	108↑	–	–	–	–	–	
	*Coelastrum* sp.	36↑	–	36↑	–	–	–	–	–	Low density*
	*Desmodesmus brasiliensis*	–	–	–	–	108↑	–	–	–	Low density
	*Desmodesmus costatogranulatus*	–	–	–	–	108↑	–	–	–	Low density
	*Dictyosphaerium* sp.	–	–	36↑	–	–	–	–	–	Low density
	*Dictyosphaerium subsolitarium*	–	–	–	–	–	108↑	–	–	Low density
	*Geminella* sp.	–	108↓	–	–	–	–	–	–	Low density
	*Gonium* sp.	–	–	–	–	–	108↑	108↑	–	SI Fig. II-B
	*Monoraphidium griffithii*	–	108↑	–	–	–	–	–	–	Low density
	*Mougeotia* sp.	–	–	–	–	–	–	108↑	–	Low density
	*Nephrochlamys* sp.	–	–	–	–	108↑	–	–	–	Low density
	*Nephrocytium* sp.	–	–	–	–	–	108↑	–	–	Low density
	*Oedogonium* sp.	–	–	–	–	–	–	–	108↑	Low density
	*Oocystis* colony	–	–	–	–	–	–	–	108↑	Low density
	*Oocystis* loose cells	–	–	–	–	–	108↓	–	–	
	*Pandorina* sp.	–	108↓	–	–	–	–	–	–	Low density
	*P. lendneri*	–	108↓	–	–	–	–	–	–	
	*S. arcuatus*	36↑	–	–	–	–	–	–	–	
	*Sorastrum* sp.	–	–	–	108↑	–	–	–	–	Low density
	*Spirogyra* sp.	–	–	–	–	–	108↑	–	–	Low density
	*Tetraedron caudatum*	–	108↑	–	–	108↑	–	–	–	Low density
	*T. minimum*	–	–	108↑	–	–	–	–	–	SI Fig. II-C
	*Tetraedron triangulare*	–	–	–	–	–	108↓	–	–	Low density
	*Volvox* (loose cells)	–	–	–	–	–	–	–	–	SI Fig. II-D
Chrysophyceae	Total abundance	–	–	–	–	–	–	–	–	SI Fig. II-E
	*Chrysococcus*	–	–	–	–	–	–	–	108↑	Low density
Cryptophyceae	Total abundance	–	–	–	–	12↓	–	–	–	SI Fig. II-F
Cyanophyta	Total abundance	–	–	–	–	–	–	–	–	SI Fig. III-A
	*Anabaena* sp.	–	–	–	108↓	36↓	–	–	–	Figure 3h
	*Snowella* sp.	–	–	–	108↑	–	–	–	–	Low density
Desmidiaceae	Total abundance	–	108↓	–	–	108↓	–	–	–	SI Fig. III-C
	*Cosmarium crenulatum*	–	–	36↓	–	–	–	–	–	Low density
	*C. formosulum*	–	–	–	–	–	–	–	108↑	Low density
	*C. pachydermum var. aethiopicum*	108↑	–	–	–	–	–	–	–	Low density
	*C. polygonum*	–	108↓	–	–	–	36↓	–	–	SI Fig. III-D
	*C. tetraophthalmum*	108↓	–	–	–	–	–	–	–	Low density
	*C. turpinii*	–	–	108↑	–	–	–	–	–	Low density
	*Gonatozygon brebissonii*	–	–	–	–	–	–	–	108↓	Low density
	*Staurastrum* spp.	–	–	–	–	–	108↓	–	–	
	*Staurastrum tetracerum*	–	–	36↓	–	–	–	–	–	Low density
Diatomeae	Total abundance	–	–	–	–	–	–	108↑	–	SI Fig. III-E
	Achnanthidiaceae	–	–	–	36↓	–	–	–	–	
	*Fragilaria* sp.	–	–	–	–	–	–	108↑	–	Low density*
	Pennales	–	–	–	–	–	108↑		–	
	*Rhopalodia gibba*	–	–	–	–	–	108↑	108↑	–	SI Fig. III-F
Dinoflagellata	Total abundance	–	–	–	108↓	–	108↓	–	–	SI Fig. III-G
	*Peridinium* sp.	–	–	–	108↓	–	108↓	–	–	
Euglenophyceae	Total abundance	–	–	–	–	–	–	–	108↓	SI Fig. III-H
	*Trachelomonas* gr. *oblonga*	–	108↓	–	–	–	–	–	–	

This table lists all taxa presented in figures and taxa for which at least on one sampling date a statistically significant effect was observed

↓ = reduction in abundance, ↑ = increase in abundance, – = no significant effect (Williams test, *p* > 0.05). *SI* Supporting Information

* Low density means that the number of individuals per sample was on average <10 individuals/mL when the statistically significant difference was observed

PRC analysis demonstrated that metiram treatment did not explain a significant component of the variation in phytoplankton community composition (Monte Carlo permutation test *p* = 0.544). Nevertheless, statistically significant treatment-related effects could be calculated for 42 of the 109 phytoplankton taxa (not including the abundance of main taxonomic groups), although the vast majority of these taxa (37 out of 42) showed a statistical significant response on an isolated sampling day only and mostly concerned low density taxa (<10 individuals/ml). In addition, statistically significant responses related to both decreases (15 cases) and increases (27 cases) in abundance and were mostly observed in the highest treatment only (NOEC of 108 μg a.i./L) (Table 6).

The blue-green alga *Anabaena* sp. (Fig. 3h) was one of the few phytoplankton taxa that showed a clear treatment-related decline in abundance on two consecutive sampling days (day 17 NOEC = 108 μg a.i./L and day 24 NOEC = 36 μg a.i./L), followed by recovery (Table 6).

In the Supporting Information more detailed information is provided on the treatment-related responses on total abundance of the main taxonomic groups of algae, and on abundance of individual phytoplankton taxa that showed a statistical deviation on at least two consecutive sampling days, or on a single sampling day during the application period (day 3–17).

### Biomass of macrophytes

Prior to the metiram application the above-sediment macrophyte biomass was estimated to be 58.6 ± 13.4 g dry weight per enclosure (geomean ± SD; *n* = 3). At the end of the study the above-sediment macrophyte biomass in control enclosures had increased to 79.8 ± 6.8 g dry weight (geomean ± SD; *n* = 4), but no significant treatment-related effects on above-sediment biomass could be observed in the treated enclosures when compared to controls (also see Supporting Information).

### Microbial endpoints and alder leaf decomposition

**Fig. 4:**
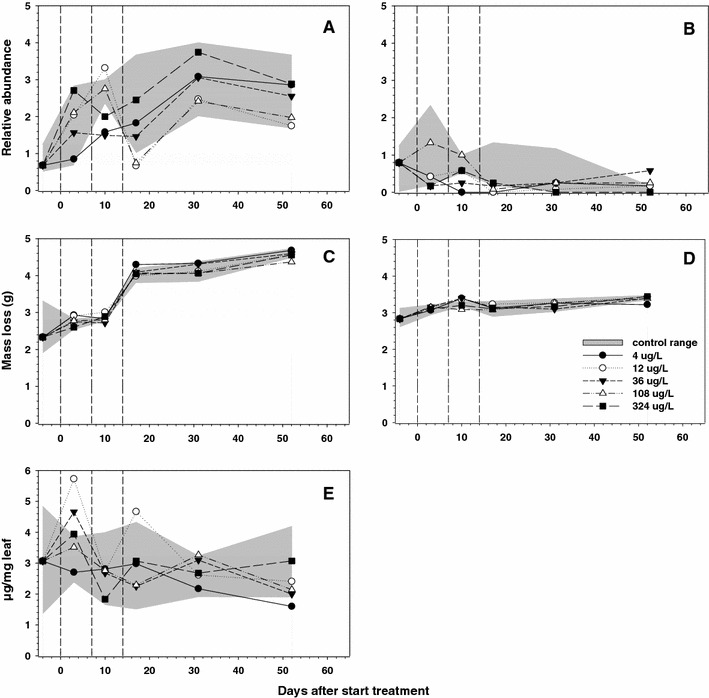
Dynamics in conidia abundance scores of aquatic hyphomycetes, and alder leaf decomposition. The *shaded area* shows the range observed in control enclosures and the mean values are presented per treatment. **a** Conidia abundance score *A.* *longissima*, **b** conidia abundance score *T. setigerum*, **c** mass loss (g dry weight) of alder leaves in coarse mesh bags, **d** mass loss (g dry weight) of alder leaves in fine mesh bags, **e** fungal biomass (μg/mg) in decomposing alder leaves. The NOECs for treatment-related responses are presented in Table 7

Based on conidia abundance, the dominant aquatic hyphomycetes on pre-conditioned alder leaf material were *Angillospora* *longissima* and *Tetracladium setigerum*. Whereas in controls the abundance score of *A. longissima* conidia generally increased during the course of the experiment (Fig. 4a), the abundance of *T. setigerum* conidia remained relatively low (Fig. 4b). For both species statistically significant treatment-related effects could not be demonstrated (William’s test, *p* > 0.05) despite the trend in lower abundance for *T. setigerum* in most enclosures that received metiram (Fig. 4b). A statistically significant effect of metiram on total fungal biomass (increase) associated with alder leaf litter could be observed on sampling day 3 only (William’s test, *p* < 0.05; NOEC 4 μg a.i./L; Table 7). This effect, however, did not show a clear concentration–response relationship (Fig. 4e). Mass loss of decomposing alder leaves increased during the experiment; mass loss in coarse mesh bags (Fig. 4c) increasing at a faster rate than mass loss in fine mesh bags (Fig. 4d). However, there was no significant treatment effect on mass loss in both types of litter bags (William’s test, *p* > 0.05).

**Table 7:** NOECs (Williams test, *p* < 0.05) in μg a.i./L (expressed in terms of nominal treatment level) for microbial endpoints and alder leaf breakdown on each sampling date in the metiram enclosure experiment

Endpoint	Day after first application								Note
	−4	3	10	17	24	31	48	59	
Conidia *A. longissima*	–	–	–	–	–	–	–	–	Figure 4a
Conidia *T. setigerum*	–	–	–	–	–	–	–	–	Figure 4b
Alder leaf mass loss (coarse)	–	–	–	–	–	–	–	–	Figure 4c
Alder leaf mass loss (fine)	–	–	–	–	–	–	–	–	Figure 4d
Fungal biomass alder leafs	–	4↑^a^	–	–	–	–	–	–	Figure 4e
Sediment bacteria									
Intensity DGGE bands	108	108	–	108	–	108	–	–	Figure 2b
OTUs	108	108	–	108	–	108	–	–	Figure 2c
Sediment fungi									
Intensity DGGE bands	–	–	–	–	–	–	–	–	
OTUs	–	–	–	–	–	–	–	–	

↑ = increase, – = no significant effect (Williams test, *p* > 0.05)

^a^Clear concentration–response relationship not observed

PRC analysis indicated that sediment bacterial community structure differed significantly between control and metiram-treated enclosures whether expressed in terms of relative band density values of the DGGE profiles (Fig. 2b; Monte Carlo permutation test *p* < 0.05) or OTUs (Fig. 2c; Monte Carlo permutation test *p* < 0.05). Given that significant differences between control and treated enclosures were present pre-application (i.e. day −4, Table 7) they cannot be attributed to the metiram treatments. PRC analyses detected no significant effect of metiram application on the sediment fungal community structure (Monte Carlo permutation test *p* > 0.05).

## Discussion

### Dissipation of metiram

In our microcosm experiment, dissipation of metiram from the water compartment was fast (overall dissipation half-life 1–6 h) and the metabolites formed were not persistent. A laboratory DT50 of 0.7 days for metiram in water–sediment systems is reported (www.eu-footprint.org). Note that this DT50 should be interpreted with caution, since metiram is a polymer less soluble in water but disintegrating fast in this compartment. The fast dissipation of metiram from the water compartment is in accordance with results of Dutch chemical monitoring programmes. Metiram was hardly ever detected in surface waters despite its frequent use in the Netherlands (www.bestrijdingsmiddelenatlas.nl).

### Responses of microbes

Despite being a fungicide, there was no evidence that metiram adversely affected the biomass, abundance or functioning of aquatic fungi. This may in part be due to the fact that the study was performed in a lentic system and aquatic fungi, in particular aquatic hyphomycetes, are more abundant and play a more important ecological role in lotic systems (Maltby 1992). The most likely explanation, however, is low exposure due to the fast dissipation of metiram in water. Note that on the plant surfaces of treated crops the exposure concentrations of metiram may be orders of magnitude higher than in the water column of our test systems.

Although on isolated sampling dates, minor differences on DGGE profiles (presence and absence of bands as well as intensity of bands) could be observed between sediment samples of controls and treated test systems, statistical analysis of the sediment microbial communities failed to show significant (fungi) or consistent (bacteria) effects of the metiram treatment. However, it is important to keep in mind the limitations of the PCR–DGGE technique in demonstrating treatment-related effects. Other studies revealed that bacterial populations that make up less than 1 % of the total community cannot be detected by PCR–DGGE (Muyzer et al. 1993; Murray et al. 1996), meaning that possible effects on low abundance populations could not be detected. However, an important question at stake is whether these low abundance populations are crucial for the ecosystem services provided. Another important limitation when dealing with a high number of samples is gel-to-gel variation, which can occur even with a well-established and standardized methodology (Powell et al. 2003; Nakatsu 2007). When dealing with complex and diverse microbial communities and a high number of samples, perfect alignment of obtained DGGE profiles is often laborious and difficult to obtain, which can mislead the analysis.

As already mentioned little information is available on the ecological impact of realistic dithiocarbamate fungicide exposures on freshwater microbial communities. Milenkovski et al. (2010) demonstrated effect of thiram and captan on denitrification, although at higher exposure concentrations (>2–3 mg a.i./L) than normally predicted for edge-of-field surface water due to normal agricultural use. Widenfalk et al. (2008) found that bacterial activity, and fungal and microbial biomass of a freshwater sediment were not affected by exposure to environmentally relevant concentrations of the fungicide captan. However, they observed significant shifts in the bacterial community composition using molecular techniques. In future studies, the use of more novel sequencing techniques may be necessary to get a better identification and understanding of the potential effects of environmentally realistic pesticide concentrations on microbial communities. For example, the combination of PCR–DGGE and sequencing techniques have been applied successfully to demonstrate impacts of different pesticides on soil microbial communities (Bending et al. 2007; Zhang et al. 2009). Furthermore, next generation sequencing-based approaches, such as 454 pyrosequencing analysis of barcoded PCR amplicons can provide information with respect to composition of microbial communities, including less abundant populations (Andersson et al. 2008; Lauber et al. 2009).

### Threshold level for community and population effects

A summary of the treatment-related responses observed in our metiram enclosure experiment is provided in Table 8. In this table the treatment-related impacts on several endpoint categories are expressed in terms of Effect Classes (see European Commission 2002; Brock et al. 2006; De Jong et al. 2008). Of all endpoints investigated, the zooplankton community and several populations of Rotifera and Copepoda showed the clearest treatment-related response due to metiram application. The lowest-observed-effect concentration (LOEC) observed for the zooplankton community was 108 μg a.i./L (Effect class 3A), while at the population level the lowest LOEC for a treatment-related decline in abundance was 36 μg a.i./L. This LOEC, however, was observed on isolated sampling days only (Effect class 2, during application period; Effect class 1–2, after application period) and the LOECs for more prolonged effects were 108 μg a.i./L (Effect class 3A) for rotifer populations and 324 μg a.i./L (Effect class 3A) for the decline in Copepoda abundance. In contrast to zooplankton, consistent prolonged treatment-related effects on macroinvertebrate endpoints were only observed for Dytiscidae larvae (Insecta) at the end of the experiment (Effect class 3A–4; LOEC = 324 μg a.i./L). There was a small decline in phytoplankton taxon richness at the highest treatment level during the application period (Effect class 2) and treatment-related effects (increases and decreases in abundance) were observed for several phytoplankton taxa, although they were usually small in magnitude and/or observed on isolated sampling dates (Effect classes 1–2 or 2). The lowest LOEC for a phytoplankton taxon that showed a treatment-related decline on consecutive sampling days (Effect class 3A) was 324 μg a.i./L (Cyanophyta, *Anabaena* sp.). There was no evidence of treatment-related effects on macrophytes, leaf decomposition or microbial endpoints and hence the threshold level of effects based on the NOECs/LOECs of the most sensitive populations (Rotifera) in our metiram microcosm study is 12–36 μg a.i./L.

**Table 8:** Summary of the community and population level effects observed in enclosures treated with metiram on basis of Effect Classes (see European Commission 2002; Brock et al. 2006; De Jong et al. 2008)

Endpoint category	Treatment concentration (μg a.i./L)				
	4	12	36	108	324
*Zooplankton*					
PRC	1	1	1	3A	3A
Taxa richness	1	1	1	1	2↓
Rotifera	1	1	2↓; 1–2↑	3A↓; 1–2↑	3A↓; 1–2↑
Copepoda	1	1	1–2↓	1–2↓	3A↓
Cladocera	1	1	1	1	1–2↑
Ostracoda	1	1	1	1	1–2↑
*Macroinvetebrate*					
PRC	1	1	1	1	1
Taxa richness	1	1	1	1	2↓
Crustacea	1	1	1	1–2↑	1–2↑
Insecta	1	1	1	2↓:	2↓; 3A-4↑
Hirudinea	1	1	1	1	1–2↑
Mollusca	1	1	1	1	1–2↓
*Phytoplankton*					
PRC	1	1	1	1	1
Taxa richness	1	1	1	1	2↓
Chlorophyll *a*	1	1	1	1	1–2↓
Chlorophyta	1	1	1	1–2↑	2↓; 3A↑
Chrysophyceae	1	1	1	1	1–2↑
Cryptophyceae	1	1	1–2↓	1–2↓	1–2↓
Cyanophyta	1	1	1	1–2↓	3A↓; 1–2↑
Desmidiaceae	1	1	1	1–2↓	1–2↓↑
Diatomeae	1	1	1	2↓	2↓; 3A↑
Dinoflagellata	1	1	1	1	2↓
Euglenophyceae	1	1	1	1	2↓
*Macrophytes*	1	1	1	1	1
*Microbes*					
Fungal biomass on alder leaves	1	1	1	1	1
Hyphomycetes on alder leaves	1	1^#^	1^#^	1^#^	1^#^
Leaf decomposition	1	1	1	1	1
DGGE profile sediment bacteria				1	1(–3A)*
DGGE profile sediment fungi				1	1

For each endpoint category the most sensitive measurement endpoint was selected that showed a positive or negative treatment-related response

*1* effects could not be demonstrated, *1–2* slight and transient effects on an isolating sampling in the post-exposure period, *2* observed effect on a single sampling during or immediately after the exposure period, *3A* pronounced effects on consecutive samplings, and total period of effects <8 weeks, *4* pronounced effects (at the end of the experiment) and study too short to demonstrate recovery within 8 weeks, *↓* = decrease, ↑ = increase

^#^Statistically significant increase observed on day 3 but clear concentration–response relationship absent

* Statistical differences observed but deviations from controls were minor and already occurred in the pre-treatment period

We are not aware of other aquatic micro/mesocosm experiments conducted with the fungicide metiram that have been published in the open literature, and thus allowing comparisons of population/community level effects with our study. However, micro/mesocosm experiments have been performed with other dithiocarbamate fungicides for regulatory purposes (not published in the open literature) in which Rotifera populations were also amongst the most sensitive populations. Aquatic model ecosystem experiments with other types of fungicides, such as carbendazim (Van den Brink et al. 2000), pentachlorophenol (Willis et al. 2004), triphenyltin (Roessink et al. 2006) and fluazinam (Van Wijngaarden et al. 2010) also demonstrated that Rotifera are relatively sensitive, but in these studies other groups of invertebrates (e.g. Copepoda, Cladocera, Turbellaria, Oligochaeta, Mollusca) were equally or somewhat more sensitive than rotifers. In contrast, Rotifera are hardly reduced in abundance by strobilurin fungicides (e.g. azoxystrobin; Gustafsson et al. 2010).

### Comparison of microcosm and laboratory toxicity test results

For metiram, Maltby et al. (2009) calculated a median HC5 (=hazardous concentration to 5 % of the tested species) of 40 μg a.i./L on basis of a species sensitivity distribution curve constructed with acute toxicity data for aquatic algae and invertebrates. The ecological threshold level found in our metiram enclosure study is fully in accordance with the observation of Maltby et al. (2009) that population and ecosystem level effects in aquatic micro/mesocosms repeatedly exposed to a pesticide with high certainty do not occur at peak concentrations that are lower than the median acute HC5 divided by an assessment factor of 3. The database used by Maltby et al. (2009) on which this relationship between HC5 values and threshold values from micro/mesocosm experiments is based contains several insecticides, herbicides and fungicides, to which metiram can now be added.

### Direct and indirect population-level effects and community metabolism endpoints

On basis of the available acute laboratory toxicity tests for aquatic organisms and metiram it was expected that populations of algae would be at least as sensitive than invertebrates (Maltby et al. 2009). In our study, however, zooplankton populations of Rotifera, and to a lesser extend Copepoda, were the most sensitive, while reductions in abundance of phytoplankton taxa were limited and less pronounced. In part this may be explained by the fact that the database of laboratory toxicity tests with aquatic invertebrates and metiram did not include Rotifera, but also the combination of rapid growth and recovery rates of algae and sampling frequency could have masked possible effects.

The short-term, but treatment-related decreases in several phytoplankton groups coupled with the small increase in electronic conductivity and alkalinity and the minor decrease in pH and DO during and/or immediately after the application period, suggest the occurrence of some treatment-related effects on primary producers. Effects of pesticides on the DO–pH–EC–Alkalinity syndrome via direct or indirect effects on the photosynthesis and metabolism of algae have been reported frequently (e.g. Brock et al. 1993; Van Wijngaarden et al. 2010). The fact that this treatment-related decrease in primary productivity did not cause prolonged treatment-related declines in abundance of phytoplankton populations may to some extent be explained by the compensation of reduced phytoplankton grazing by Rotifera and Copepoda (interplay of direct and indirect effects) or by the sublethal nature of the metiram effects on algae.

Compensation of direct toxic effects on phytoplankton due to toxicant-induced reduced grazing by zooplankton has been observed in aquatic model ecosystem experiments treated with other broad spectrum fungicides (e.g. Van den Brink et al. 2000; Roessink et al. 2006) and biocides (Fliedner et al. 1997; Jak et al. 1998) and in microcosms treated with an insecticide–herbicide mixture (Van den Brink et al. 2009). The observed short-term increases in population densities of several macroinvertebrate taxa in our microcosm experiment may also be due to shifts in species interactions caused by direct toxic effects of metiram. Indirect effects in toxicant-stressed aquatic micro-/mesocosms are reported to be more pronounced if the toxicant eliminates key species that do not recover rapidly, causing shifts in species interactions in the same (release of competition) or adjacent trophic level (release of grazing or predation) (see e.g. Baird et al. 2001; Fleeger et al. 2003; Relyea and Hoverman 2006; Clements and Rohr 2009). This apparently is not the case in our metriram enclosure study.

### Ecological recovery

In our study we found fast recovery of all affected measurement endpoints (effect period < 8 weeks). According to Brock et al. (2008), and literature cited therein, recovery of affected populations from pesticide-stress in aquatic ecosystems may be rapid if the following conditions apply: the pesticide is not persistent and the exposure regime is short-term; the generation time of the populations affected is short; population reductions are only partial and/or pesticide-resistant life stages (e.g. eggs and ephyppia) are present (internal recovery); there is a ready supply of propagules of eliminated populations through active immigration by swimming or flying organisms or through passive immigration by e.g. wind and water transport (external recovery). Obvious explanations for the fast ecological recovery in our microcosm experiment are (1) the short-term exposure to metiram (overall dissipation DT50 1–6 h) despite its repeated application and (2) the short generation time of the sensitive populations affected. According to Barnthouse (2004) the reported mean generation time of Rotifera is 8 days with a range of 6 to 35 days.

## Conclusions

In field enclosures that simulated an aquatic community of shallow drainage ditches, the dissipation of metiram from water was very fast (overall dissipation half-life 1–6 h). A few days after the third and last weekly metiram application (nominal concentrations of 4–324 μg a.i./L), the metabolites EU and ETU were measured in water above their detection limit, but these substances were not persistent. Amongst the large number of biological endpoints measured, the zooplankton community, and Rotifera populations in particular, showed clear treatment-related effects, followed by fast recovery (within 8 weeks after the first application). Treatment-related effects on phytoplankton and macroinvertebrates were minor and transient. There was no evidence that metiram application adversely affected alder leaf breakdown, fungal biomass and abundance of aquatic hyphomycetes on leaf litter. In addition, consistent treatment-related effects on the microbial community in the sediment compartment were not observed. The threshold level of effects based on the NOECs of the most sensitive populations (Rotifera) in our metiram microcosm study is 12–36 μg a.i./L.

## Electronic supplementary material

Below is the link to the electronic supplementary material.

Supplementary material 1 (DOCX 908 kb)
