# Supplementary material for: Effects of the fungicide metiram in outdoor freshwater microcosms: responses of invertebrates, primary producers and microbes
Source: Ecotoxicology. 2012 May 4;21(5):1550–69. doi: 10.1007/s10646-012-0909-0 (PMC3377896; doi:10.1007/s10646-012-0909-0)
Supplement: Supplementary file 1 — Supplementary material 1 (DOCX 908 kb) [file 10646_2012_909_MOESM1_ESM.docx]

**Supporting Information:** Lin *et al*. Effects of the fungicide metiram in outdoor freshwater microcosms: Responses of invertebrates, primary producers and microbes

*Weather conditions during the experiment*

During the metiram enclosure experiment (29 July 2011 – 9 October 2011) mean daily air temperatures ranged between 12.1 and 22.4 ºC. Weather conditions during the day of the first application (10^th^ of August) can be characterised as moderately warm (mean air temperature 10 cm above soil 18.2 ºC), partly clouded (265 minutes of sunshine) and relatively dry (3.0 mm of rainfall). During the second application (17^th^ of August) the mean air temperature was 16.3 ºC, the sky was cloudy (0 minutes of sunshine) and there was 1.3 mm of rain. The temperature on the third application date (24^th^ of August) was 17.0 ºC and it was generally sunny (571 minutes of sunshine), but a shower resulted in 5.5 mm of rainfall.

*Physico-chemical measurements*

|  |   **C**  **A**  **D**  **B** |  |
| --- | --- | --- |
|  |  |  |
|  | **Days after start treatment** | |

**SI Figure I**. Dynamics in electronic conductivity (panel A), pH (panel B), dissolved oxygen (panel C) and alkalinity (panel D) in water of enclosures of the metiram experiment. The three dotted vertical lines in the panel indicate the moments of metiram application. The NOECs for treatment-related responses are presented in Table 3.

*Treatment-related responses on phytoplankton*

Overall, the total abundance of Chlorophyta was high and relatively constant in all enclosures and metiram application did not result in a treatment-related effect (SI Figure II-A; Table 6 in main document). Although statistically significant responses could be observed for 24 Chlorophyta taxa, they were only observed on consecutive sampling days for one taxon (*Gonium* sp.). *Gonium* sp. occurred in low densities (SI Figure II-B), but treatment-related increases in abundance were observed on days 31 and 48 at the highest treatment level (Table 6 in main document). During, or immediately after, the application period (days 3 -17) three Chlorophyta taxa (*Geminella* sp., *Pandorina* sp. and *Phacotus lendneri*) exhibited a treatment-related decrease on a single sampling date at the highest treatment level (Table 6 in main document). Over the same time period, seven Chlorophyta taxa showed an increase in abundance on an isolated sampling day: Chlorophyta (loose cells), *Tetraedron minimum* (SI Figure II-C), *Coelastrum* sp., *Dictyosphaerium* sp., *Monoraphidium griffithii*, *Sorastrum* sp. and *Tetraedron caudatum* (Table 6 in main document). Since the taxon *Volvox* (loose cells) dominated the phytoplankton community, the dynamics in abundance for this taxon are shown in SI Figure II-D. Although a statistically significant effect following metiram application could not be demonstrated due to the high variability in abundance between control replicates, a trend of a short-term decline in *Volvox* densities could be observed at the two highest treatment levels (SI Figure II-D).

Overall, the total abundance of Chrysophyceae (SI Figure II-E) and Cryptophyceae (SI Figure II-F) was relatively low and statistically significant treatment-related effects were only observed for total Cryptophyceae on day 24 (NOEC = 12 µg a.i./L) and for one low density Chrysophyceae taxon, *Chrysococcus*, on day 59 (NOEC = 108 µg a.i./L) (Table 6 of main document).

| **Abundance/mL** |   **C**  **A**  **E**  **D**  **B**  **F** |  |
| --- | --- | --- |
|  |  |  |
|  |  |  |
|  | **Days after start treatment** | |

**SI Figure II:** Dynamics in population abundance of phytoplankton taxa in the different treatments of the metiram enclosure experiment. The shaded area shows the range observed in control enclosures and the geometric mean values are presented per treatment. A: Total Chlorophyta; B: *Gonium* sp. (Chlorophyta); C: *Tetraedron minimum* (Chlorophyta); D: Volvox loose cells (Chlorophyta); E: Total Chrysophyceae; F: Total Cryptophyceae.

The total abundance of Cyanophyta was high and relatively constant in all enclosures and metiram application did not result in a significant treatment-related effect (SI Figure III-A; Table 6 of main document). The blue-green alga *Anabaena* sp. (SI-Figure III-B) showed a treatment-related decline in abundance on two consecutive sampling days (day 17 NOEC = 108 µg a.i./L and day 24 NOEC = 36 µg a.i./L), followed by recovery (Table 6 of main document). Another blue-green alga, *Snowella* sp., also showed a statistically significant treatment-related response on day 17 (NOEC of 108 µg a.i./L), but here abundance increased (Table 6 of main document).

The total abundance of Desmidiaceae in the enclosures was relatively constant during the experimental period; however, a small treatment-related decrease was apparent from day 3 up until day 24 (SI Figure III-C) and statistically significant decreases in total abundance were observed on days 3 and day 24 (NOEC = 108 µg a.i./L) (Table 6 of main document). Statistically significant responses could be calculated on a single sampling date for nine Desmidiaceae taxa, which, with the exception of *Cosmarium polygonum* (SI Figure III-D), all occurred in low densities (on average < 10 individuals/ml). Significant declines in the abundance of *Cosmarium polygonum* were apparent on day 3 and day 31 (Table 6 of main document), but the day 31 NOEC of 36 µg a.i./L is considered less valid due to a poor concentration-response relationship (SI Figure III-D). During the application period a NOEC of 36 µg a.i./L (day 10; decrease) was observed for two low density taxa, *Cosmarium crenulatum* and *Staurastrum tetracerum* (Table 6 of main document).

The total abundance of Diatomeae was relatively high and gradually declined during the course of the study (SI Figure III-E), although a small treatment-related increase in total diatom abundance was calculated for day 48 (NOEC = 108 µg a.i./L) (Table 6 of main document). Statistically significant increases in abundance were observed for *Rhopalodia gibba* on two consecutive sampling days (SI Figure III-F, day 31 – 48) and for two other taxa (*Fragilaria*, Pennales) on a single occasion during later phase of the study; in all cases the NOEC was 108 µg a.i./L (Table 6 of main document). During the metiram application period a NOEC of 36 µg a.i./L (decrease) could be calculated for Achnanthidiaceae on day 17 (Table 6 of main document).

Densities of Dinoflagellata were generally low (SI Figure III-G) and statistically significant declines due to metiram application were observed on sampling days 17 and 31 (NOEC = 108 µg a.i./L). On the same sampling dates the same NOEC was observed for the taxon *Peridinium* sp. (Table 6 of main document).

| **Abundance/mL** |   **A** |   **B** |
| --- | --- | --- |
|  |  |   **D**  **C** |
|  |   **F**  **E** |  |
|  |   **H**  **G** |  |
|  | **Days after start treatment** | |

**SI Figure III:** Dynamics in population abundance of phytoplankton in the different treatments of the metiram enclosure experiment. The shaded area shows the range observed in control enclosures and the geometric mean values are presented per treatment. A: Total Cyanophyta; B: *Anabaena* sp. (Cyanophyta); C: Total Desmidiaceae; D: *Cosmarium polygonum* (Desmidiaceae); E: Total Diatomeae; F: *Rhopalodia gibba* (Diatomeae); G: Total Dinoflagellata; H: Total Euglenophyceae.

The abundance of Euglenophyceae was relatively constant throughout the study, but showed a decline on the last sampling date (SI Figure III-H) and a NOEC of 108 µg a.i./L (decline) could be calculated. Within this taxonomic group, a statistically significant decline was observed for *Trachelomonas* gr. *oblonga* on day 3 with a NOEC of 108 µg a.i./L (Table 6 of main document).

*Biomass of macrophytes*

|  |
| --- |
| **Treatment level** |

**SI Figure IV**. Above sediment dry weight biomass (mean + SD) of macrophytes in the enclosures at the end of the metiram experiment.
